# Near-Ultraviolet Circular Dichroism and Two-Dimensional Spectroscopy of Polypeptides

**DOI:** 10.3390/molecules26020396

**Published:** 2021-01-13

**Authors:** Francesco Segatta, David M. Rogers, Naomi T. Dyer, Ellen E. Guest, Zhuo Li, Hainam Do, Artur Nenov, Marco Garavelli, Jonathan D. Hirst

**Affiliations:** 1 Dipartimento di Chimica Industriale “Toso Montanari”, Universita’ degli Studi di Bologna, Viale del Risorgimento, 4, I-40136 Bologna, Italy; francesco.segatta2@unibo.it (F.S.); artur.nenov@unibo.it (A.N.); marco.garavelli@unibo.it (M.G.); 2 School of Chemistry, University of Nottingham, University Park, Nottingham NG7 2RD, UK; david.rogers@nottingham.ac.uk (D.M.R.); naomi_414@hotmail.com (N.T.D.); ellen.guest@nottingham.ac.uk (E.E.G.); 3 School of Pharmaceutical Sciences, Jilin University, Changchun 130021, China; zhuoli198602@gmail.com; 4 Department of Chemical and Environmental Engineering, University of Nottingham Ningbo China, Ningbo 315100, China; 5 New Materials Institute, University of Nottingham Ningbo China, Ningbo 315042, China; hainam.do@nottingham.edu.cn

**Keywords:** electronic structure, computational spectroscopy, protein, aromatic chromophores

## Abstract

A fully quantitative theory of the relationship between protein conformation and optical spectroscopy would facilitate deeper insights into biophysical and simulation studies of protein dynamics and folding. In contrast to intense bands in the far-ultraviolet, near-UV bands are much weaker and have been challenging to compute theoretically. We report some advances in the accuracy of calculations in the near-UV, which were realised through the consideration of the vibrational structure of the electronic transitions of aromatic side chains.

## 1. Introduction

Energy transfer in photosynthetic macromolecular complexes [1], electronic dynamics in semi-conductors, and photochemical reactions [2] are just some of the cases where coherent two-dimensional electronic spectroscopy [3,4] is providing new insights. Its application in the UV region (2DUV) offers a powerful tool for probing the photophysics and photochemistry of biomolecular systems such as proteins [5], as the spectra reflect the interactions among aromatic side chain groups, which is of interest in the context of protein structure and stability [6] as well as electron and energy transfer processes [7]. The interpretation of complex and often congested spectra benefits considerably from computational modelling. Several theoretical studies have predicted the two-dimensional spectra of proteins in the far-ultraviolet [8,9,10] and in the near-ultraviolet [11,12,13], for example, characterising the signals expected for amyloid fibrils. Conti et al. [14] have recently provided a comprehensive overview of the state-of-the-art theoretical methods and their application to ultrafast spectroscopy.

Conventional (one-dimensional) techniques, such as electronic absorption spectroscopy, linear dichroism and electronic circular dichroism (CD) spectroscopy are less information-rich than 2DUV but much easier to measure and to compute for biomolecules from first principles. Nevertheless, some of the underlying computational framework is common across all techniques, and in particular, CD spectroscopy is widely used to study proteins [15]. CD spectra have both positive and negative peaks, arising from the differential absorption of left and right circularly polarised light. In the far-UV, the CD spectra of proteins reflect the secondary structure content. In the near-UV, the spectra are sensitive to the separation and relative orientation of the aromatic side chains [16].

Bovine pancreatic trypsin inhibitor (BPTI) is a widely studied protein in the context of folding and spectroscopy [17]. Of the 58 residues that comprise BPTI, a high proportion is aromatic and so contribute to spectroscopic signals in the near-UV. First principles calculations of its near-UV CD spectrum show an elevated sensitivity to the protein’s precise conformation [18,19], which arises from the influence of the environment of two particular tyrosine residues on the angle between the electric and magnetic transition dipoles. In particular, an angle of less than 90° gives a positive rotational strength and an angle greater than 90° gives a negative rotational strength.

Small peptide systems, e.g., cyclic dipeptides, provide useful models to deepen the understanding of the mechanisms that govern the electronic structure of interacting chromophores [20,21,22,23]. Snow et al. [24] investigated cyclo(l-Ala-l-Tyr) and cyclo(l-Tyr-l-Tyr). They suggested conformations based on energy minimisation and the comparison of computed and experimental near-UV CD. Madison et al. [25] reported the spectrum of cyclo(l-Pro-l-Tyr) in water, acetonitrile, and chloroform. There is some sensitivity of the CD spectrum to solvent, which probably is due to different conformational populations. In acetonitrile, the L_b_ band of cyclo(l-Pro-l-Tyr) is positive; it was suggested that this corresponds to a conformation of side chain dihedral angles (χ_1_/χ_2_ = 60°/90° or 200°/60°, where χ_1_ and χ_2_ are defined by rotations round the C_α_-C_β_ and C_β_-C_γ_ bonds, respectively, of the side chain). Madison et al. [25] suggest that the L_b_ band acquires optical activity largely through interaction with amide ππ* states. Several conformations are possible, and comparison between calculated and experimental CD spectra suggested that an extended conformation was more (but not exclusively) populated than a folded conformation. Li and Mukamel [26] studied cyclo(l-Pro-l-Tyr). Cyclic dipeptides containing proline have a stable boat configuration. Arachchilage et al. [27] reported a boat geometry of cyclo(l-Pro-l-Tyr) optimised at the B3LYP/cc-pVTZ level. Nenov et al. [28,29] have presented an efficient approach to reduce the computational cost of accurate simulations of 2DUV spectra of amino acids with aromatic side chains and their dimers. In a series of studies on small polypeptides, they have demonstrated the appearance of off-diagonal signatures in stacked aggregates, which can be selectively filtered out by using combinations of polarised pulse set ups. Recent combined theoretical (DFT calculations) and experimental (resonance-enhanced multiphoton ionisation and double resonance infrared ultraviolet) spectroscopic studies have examined jet-cooled cyclic dipeptides in gas phase. BenNasr et al. [30] found that the conformational landscape of cyclo(l-Tyr-l-Tyr) strongly differs from that of its diastereomer cyclo(l-Tyr-d-Tyr). The same techniques were employed to study the conformational landscape of cyclo(l-Tyr-l-Pro) and cyclo(l-Tyr-d-Pro) [31], where the simulation of the vibronic pattern of the S_0_-S_1_ transition was necessary to assign the observed spectra to either folded or extended conformations.

Recently, we have incorporated the vibrational structure of aromatic electronic excitations into exciton calculations of the near-UV CD spectra of proteins [18,19,32]. The approach and associated parameters build on earlier ab initio parameters [33] that have been assessed on a range of proteins and give good agreement between the computed and experimental near-UV CD spectra. The computed spectra show an interesting level of sensitivity to the precise protein conformation. In this study, we apply this methodology to calculate CD spectra for the cyclic dipeptides cyclo-Pro-Tyr (cPY) and cyclo-Tyr-Tyr (cYY) focusing on the spectral features of the Tyr and Phe residues in the near-UV. Next, we present 2D near-UV spectra and discuss the sensitivity of this technique. Finally, we demonstrate that CD and 2D near-UV calculations are tractable for BPTI, a 58-residue protein with four Tyr and four Phe residues. The spectral features are discussed in terms of the transitions in the Tyr and Phe residues. We consider the influence of conformational dynamics on the computed spectra of BPTI, including the impact of the fluctuating electrostatic environment.

## 2. Results and Discussion

Phenol in the gas phase has four peaks in its absorption spectrum [34]. We have summarised some of the relevant experimental and theoretical data in Table 1. Limão-Vieira et al. [35] found for gas-phase phenol that EOM-CCSD calculations generally overestimated transition energies by about 0.3 eV compared to experiment, and that for TD-DFT (LC-ωPBE/aug-cc-pVTZ + R), the difference was typically ≈0.5 eV. The pioneering work of Mukamel and co-workers [36] simulated the two-dimensional electronic spectroscopy of phenol based on transition dipole moments and excitation energies computed using multi-configurational methods. This laid the groundwork for calculations of the two-dimensional electronic spectra of more complex systems [28,37].

### 2.1. Cyclic Dipeptide

#### 2.1.1. Circular Dichroism

**Figure 1:**
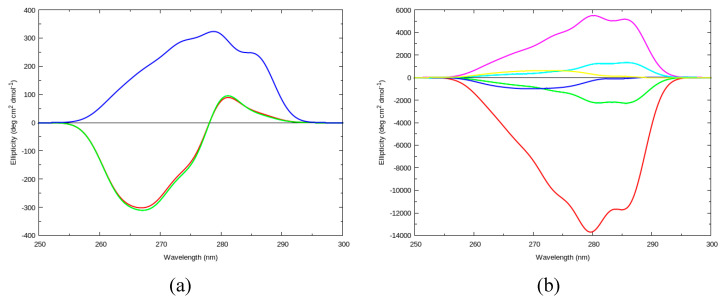
(**a**) Computed near-UV circular dichroism (CD) spectra for cPY_60 (red line), cPY_180 (green line), and cPY_300 (blue line); (**b**) Computed near-UV CD spectra for cYY_60_60 (red line), cYY_180_60 (green line), cYY_180_180 (blue line), cYY_300_60 (magenta line), cYY_300_180 (cyan line), and cYY_300_300 (yellow line). Spectra computed using BB and VSC parameters. CD spectra from rotational strength line spectra convoluted with a Gaussian of full-width half-maximum (FWHM) of 4.0 nm. BB: two amide backbone transitions, nπ* and ππ*, VSC: vibrational fine-structure in the L_b_ state of Tyr along with its vertical transitions L_a_, B_a_ and B.

CD spectra for the cyclic dipeptides cPY and cYY were computed using DichroCalc with parameters to describe two amide backbone transitions, nπ* and ππ* (BB), and vibrational fine-structure in the L_b_ state of Tyr along with its vertical transitions L_a_, B_a_ and B_b_ (VSC). There are two relevant vibrational modes [18]: a ring breathing mode (which we label *u*) with a frequency of 800 cm^−1^ and a C-H bending mode of the C-H bonds of the ring (which we label *v*) with a frequency of 1250 cm^−1^. The computed near-UV CD spectra for each of the three cPY conformers, corresponding to those considered in early studies [24,25], are shown in Figure 1a. The cyclic dipeptides are named according to their χ_1_ dihedral angles(s) for the Tyr residue(s).

cPY_60 and cPY_180 have nearly identical spectra, which are characterised by a couplet centred at 278 nm. The spectrum for cPY_300 is predicted to have positive ellipticity in the near UV. The Tyr transitions to L_b_(*u*_0_, *v*_0_), L_b_(*u*_1_, *v*_0_), L_b_(*u*_0_, *v*_1_), L_b_(*u*_2_, *v*_0_), L_b_(*u*_0_, *v*_2_), L_b_(*u*_1_, *v*_1_), L_b_(*u*_3_, *v*_0_), L_b_(*u*_2_, *v*_1_), and L_b_(*u*_4_, *v*_0_) were considered. These transitions give rise to the CD spectra in the near UV for the cPY conformers. For example, for cPY_300, the intense peak at 279 nm is due to the L_b_(*u*_1_, *v*_0_) transition. Comparing the CD spectra of cyclo-L-Ala-L-Tyr reported by Snow et al. [24] (presented in Figures 3 and 4 of ref. [24]) with the spectra in Figure 1a suggests that the most likely conformer for cPY is the cPY_300 conformation, as this conformation has a positive CD signal in the near-UV region.

The computed near-UV CD spectrum for each of the six cYY conformers are shown in Figure 1b. Two of the conformers have distinct spectra: cYY_60_60 and cYY_300_60. For cYY_60_60, the intense negative peak at 279 nm is due to the L_b_(*u*_1_, *v*_0_) transition (279.6 nm). This transition is also responsible for the intense positive peak at 279 nm for cYY_300_60. The remaining four conformers of cYY have less intense bands in their predicted near-UV CD spectra than for cYY_60_60 and cYY_300_60. They can be grouped into two pairs, that are characterised as having nearly mirror images of the other of the pair: cYY_180_60 (negative bands) with cYY_300_180 (positive bands), and cYY_180_180 (negative bands) with cYY_300_300 (positive bands). For both pairs, the conformer with the negative bands has the greater absolute ellipticity of the pair. Comparison of the CD spectra reported by Snow et al. [24] (in Figure 6 of ref. [24]) with the spectra in Figure 1b suggests that the most likely conformer for cYY is the cYY_300_60 conformation, because this conformation has the largest, positive CD signal in the near-UV region.

#### 2.1.2. 2DUV

**Figure 2:**
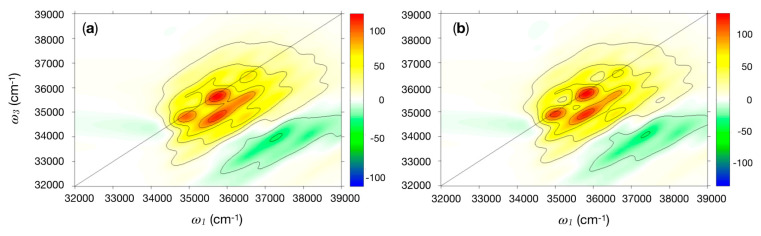
Two-dimensional electronic spectroscopy in the UV region (2DUV) spectrum in the near UV for (**a**) cPY_300; (**b**) cYY_300_60. Plots of ω_3_ (cm^−1^) versus ω_1_ (cm^−1^) for the real part of the signal. Polarisation of the four pulses is xxxx. The intensity has been renormalised to the number of Tyr (i.e., maps in (**b**) has been divided by 2).

The SPECTRON program was employed to compute the 2DUV spectra for the nine conformations of the cyclic dipeptides. The same parameter sets (BB and VSC) used for the CD calculations were here employed. The Tyr vibronic structure was modeled only with the 800 cm^−1^ mode, as the coupling to the 1250 cm^−1^ mode was found to be negligible. The coupling was modeled via a spectral density centered at the frequency of the mode and having reorganisation energy of ca. 990 cm^−1^. Results for cPY_300 and cYY_300_60, the most likely conformations, are considered in more detail. In the latter, the Tyr–Tyr electronic coupling, negligible with respect to the system-bath coupling, was set to zero (i.e., the two Tyr residues will likely not form excitons). Figure 2a displays the real signal of the quasi-absorptive 2DUV spectrum for the cPY_300 conformer. The plots are nearly identical for the real and the imaginary signal for each conformer when the all-parallel polarisation set up (labeled as xxxx) is employed. The strong transitions on the diagonal are L_b_(*u*_0_, *v*_0_) (34,965 cm^−1^), L_b_(*u*_1_, *v*_0_) (35,765 cm^−1^), and L_b_(*u*_2_, *v*_0_) (36,565 cm^−1^). These three transitions clearly dominate the spectra on the diagonal, while the expected vibronic cross-peak pattern appears in the off-diagonal region.

**Figure 3:**
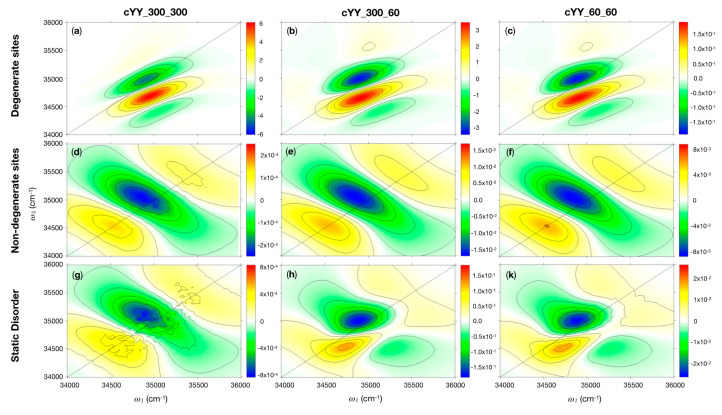
2DUV spectra with xxxx−3xxyy polarisation for cYY_300_300, cYY_300_60, and cYY_60_60 conformers are compared in the case of degenerate sites and, consecutively, strong wave function mixing (**a**–**c**), for non-degenerate sites (very weak/null wave function mixing) (**d**–**f**), and for static disordered (a variety of wave function mixing configurations in the ensemble) spectra (**g**–**k**). The intensity has been renormalised to the number of Tyr, and, in the case of static disorder spectra, also for the number of snapshots (500).

Both panels in Figure 2 are dominated by the vibronic progression of the 800 cm^−1^ Tyr ring-breathing mode and do not display any significant difference. Simulations on the other systems (not shown) reveal the same pattern presented in Figure 2. Thus, it would be extremely hard (or impossible) to distinguish between Tyr-containing peptides with a standard (parallel polarised) 2D experiment. Nonetheless, it is possible to differentiate the two systems by considering combinations of pulse polarisations, such as xxxx–3xxyy or xyxy–xyyx [39], which can unveil more subtle spectral features. The cPY signals (not reported) are completely cancelled by this polarisation combination due to the presence of a single Tyr residue, while the cYY exhibit a residual signal when the Tyr are coupled. In Figure 3, we report the results obtained by employing the xxxx–3xxyy polarisation combination for three cYY conformers. The proper treatment of the coupling between the Tyr residues requires considering the vibronic Hamiltonian explicitly. Since this is not currently available in SPECTRON, we limit our analysis on the first vibronic peak of the spectrum, which corresponds to the coupling between L_b,_(*u*_0_) transitions (see Methods in Section 3 for more details). Experiments could also focus on this transition by employing an appropriate pulse shape centered around 34,965 cm^−1^. Notably, characteristic peak patterns may be identified for different cYY conformers. The simulated 2DUV signals can be rationalised in terms of Tyr–Tyr coupling strength, relative Tyr–Tyr orientation, and strength of the static disorder.

Let us consider the combination xxxx–3xxyy for two different situations in each of the three systems cYY_300_300, cYY_300_60, and cYY_60_60:

i. Degenerate site energies: the site energies are set at the Tyr vertical energy;
ii. Randomly sampled site energies (static disorder): each one of the site energies is randomly extracted from a Gaussian distribution centered at the Tyr vertical energy.

Due to the small value of the coupling (generally < 10 cm^−1^), the energetic structure of the formed exciton does not differ from that of the monomers, exhibiting the same vibronic structure. However, when the chromophores are degenerate (case i), (see Table 2, degenerate sites), even a very weak coupling between the Tyr residues can cause a strong delocalisation of the excitation (i.e., a large site mixing in the final exciton state). This leads to an xxxx–3xxyy signal, which is asymmetric with respect to the diagonal with many more features below the diagonal (see Figure 3a–c). The shape of the spectrum slightly changes by virtue of the different Tyr–Tyr coupling strength (which increases from cYY_300_300 to cYY_60_60). Finally, a dependence of the signal intensity to the angle between the Tyr transition dipole moments is also found: cYY_300_300 and cYY_300_60, with their nearly orthogonal dipoles (70°), display a more intense signal. Instead, when the dipoles are parallel (cYY_60_60, 10°), the signal is still very low in intensity. This can be rationalised considering that in sandwich-shaped or parallel-displaced configurations where the transition dipole moments of the residues are close to parallel or anti-parallel, upon exciton formation, one of the states accumulates the entire oscillator strength, while the other state becomes dark. The residual signals in polarisation combination spectra arise due to Liouville space pathways involving transitions to *both* exciton states. Thus, it is clear that despite the strong coupling, the residual signal will vanish for (anti)-parallel dipole configurations due to the dark nature of one of the involved excitons. In support of this interpretation, if one artificially sets the cYY_60_60 transition dipole moments to 70°, the intensity of the xxxx−3xxyy strongly increases (see Table 2, column Mod. cYY_60_60, and Figure S1 in the Supplementary Materials).

Based on these considerations, one would expect the strongest xxxx−3xxyy signal for systems where the dipoles have an angle around 45°, which represents a balance between a strong inter-chromophore coupling (which has the highest magnitude for parallel dipole conformations) and non-parallel Tyr dipole orientations (with an optimum for orthogonal dipole configurations) that avoid the production of dark excitons.

The discussion hereto, while being indispensable for unravelling the effect of exciton formation on the spectral line shapes, deals with a highly unrealistic situation. In a real sample, the degenerate site energies case (i) is representative only for a minority of the aggregates. Real systems are never perfectly degenerate due to the coupling with the environment, which causes their site energies to fluctuate. The site energy splitting leads to a weak mixing and the formation of non-perfectly delocalised excitons. Furthermore, in case the energy splitting is much larger than the magnitude of the coupling, the residues would not mix at all, and the aggregate would behave as a non-interacting multimer. We simulated the spectra in the second scenario by averaging over 500 snapshots introducing a static disorder with a variance of 100 cm^−1^ for all states.

The choice of the variance is rather arbitrary (although not unrealistic for the L_b_ transition of Tyr) and only serves to compare the three systems. As expected (see Table 2, static disorder), the system with the larger inter-chromophore coupling (cYY_60_60, with a coupling between the L_b_(*u*_0_) vibronic transitions of about 7 cm^−1^) experiences the largest number of snapshots where the two chromophores couple: a strong state mixing is found for nearly 10% of the randomly sampled configurations. We apply the following criterion for “strong” site mixing: the exciton wavefunctions must have coefficients between 0.71/±0.71 (perfect exciton) and 0.90/± 0.44. Instead, cYY_300_300 is virtually uncoupled. We observe the largest signal intensity of the xxxx–3xxyy combination (relative to the all-parallel polarisation) in cYY_300_60 due to the aforementioned balance between a relatively strong coupling and a 70° angle between the dipoles. Nonetheless, the signal is quite low, about 1% of the parallel polarised signal due to the generally small value of the coupling (2.6 cm^−1^). The lineshape of the xxxx–3xxyy signal obtained from the static disorder simulations (Figure 3g–k) resembles that of the non-degenerate, weakly mixing chromophores (see Figure 3d–f) for the very weakly coupled cYY_300_300 system, while it resembles an average between the weakly and strongly mixing cases for cYY_300_60 and cYY_60_60. This underlines the fact that even if strongly mixing configurations are a minority, they are more intense than weakly mixing configurations and therefore contribute significantly to the overall spectrum.

A final remark concerns the lifetime of the formed excitons that lead to strongly mixing configurations: this will be extremely short if the ratio between the Tyr–Tyr coupling and the Tyr coupling to the bath is small (as it may be in most of these cases). The latter being controlled by temperature, one may observe longer lived excitons (i.e., stronger differences in the xxxx–3xxyy signals) by lowering it.

To summarise: 2DUV maps recorded with the xxxx–3xxyy combination of polarisations are able to deliver some structural information. This signal is dependent on both the inter-Tyr coupling and the orientation of the two Tyr in the peptide. In general, these findings are directly transferable to systems with stronger coupling. For Y-containing polypeptides, the effect of exciton formation may be observed at a low enough temperature such that environment fluctuations are suppressed and do not destroy the fragile excitons formed due to the weak coupling.

### 2.2. BPTI

The BPTI polypeptide contains four Tyr and four Phe residues that contribute to the near-UV spectral window between 250 and 300 nm. In the simulations, each Tyr and Phe residue is described by two amide backbone transitions, nπ* and ππ* (BB) and four transitions in the aromatic side chain labeled L_b_, L_a_, B_a_, and B_b_ (VSC). The vibrational fine structure in the L_b_ states of Tyr and Phe is explicitly incorporated. As done for the dipeptides, Tyr is treated as strongly coupled to a mode with a frequency of 800 cm^−1^ and reorganisation energy of ca. 990 cm^−1^. The Phe L_b_ state is characterised by a significant coupling with two intra-molecular modes, at approximately 570 and 980 cm^−1^, having reorganisation energies of 300 and 660 cm^−1^, respectively. Therefore, the total reorganisation energy is 960 cm^−1^. The remaining non-aromatic residues contribute only a pair of BB transitions.

#### 2.2.1. Circular Dichroism

**Figure 4:**
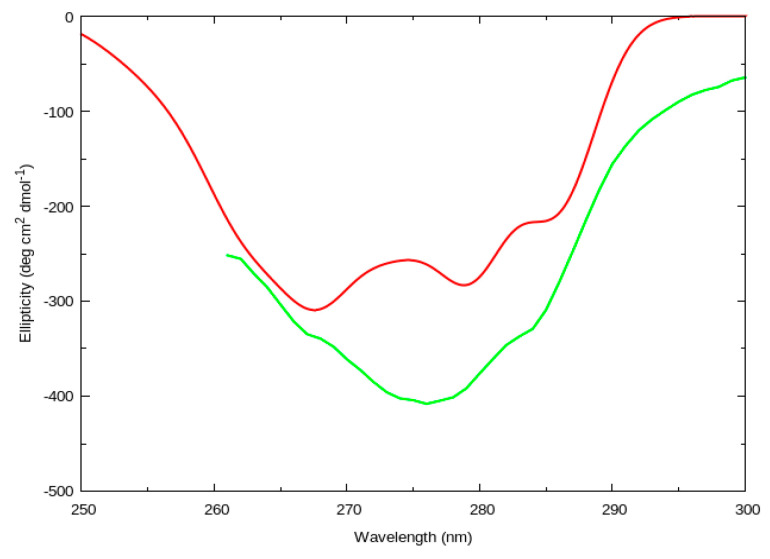
Computed (red line) and experimental (green line) [17] near-UV CD spectra for bovine pancreatic trypsin inhibitor (BPTI, PDB entry 1PIT). The computed spectrum is an average of rotational strength line spectra computed for 20 NMR models, using BB and VSC parameters for Tyr and Phe, and convoluted with a Gaussian of FWHM of 4.0 nm.

Jasim et al. [19] have studied the near-UV CD spectra of BPTI in detail, where the influence of the electrostatic environment of the protein on the CD spectra has also been considered [32]. The computed and the experimental [17] spectra are shown in Figure 4. The computed CD spectrum is an average over rotational strength line spectra computed for 20 NMR models, from Protein Data Bank (PDB) entry 1PIT, and it is convoluted with a Gaussian of FWHM of 4.0 nm.

#### 2.2.2. 2DUV

**Figure 5:**
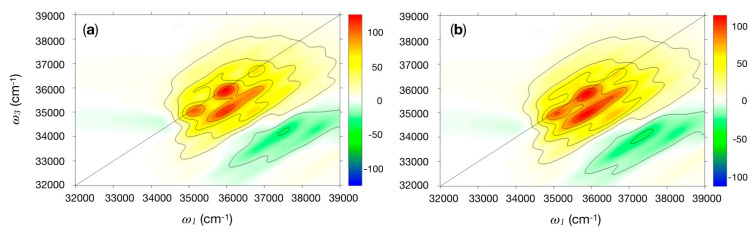
(**a**) 2DUV spectra in the near UV for BPTI with a pulse polarisation of xxxx for a single configuration with degenerate energies; 2DUV spectra in the near UV for BPTI with a pulse polarisation of xxxx (**b**) with electronic Hamiltonian with the electrostatic fluctuations (EHEF) correction. Plots of ω_3_ (cm^−1^) versus ω_1_ (cm^−1^) for the real part of the signal. The intensity has been renormalised to the number of Tyr (i.e., by a factor of 4 in BPTI). The spectrum in (**b**) was also averaged over the 200 MD snapshots.

Near-UV 2D spectra were also simulated for BPTI. Spectra with degenerate site energies are first considered (Figure 5a). As observed for the cyclic dipeptides, the signals in the spectral region between 250 and 300 nm are due to the vibrational progression of the L_b_ state of Tyr. The Phe contributes to the spectrum with a weak absorption above 37,310 cm^−1^. Studies on Phe–Tyr dimers have shown that these contributions can be enhanced by narrowband pump pulses centered at the Phe transition [40].

Next, physically founded static disorder is introduced by employing 200 snapshots from a molecular dynamics (MD) simulations and introducing site energy fluctuations in the electronic Hamiltonian with the electrostatic fluctuations (EHEF) technique. Electrostatic fluctuations in the environment can affect inter- and intra-molecular transitions [41], and these changes affect the local Hamiltonian of the chromophore. Fluctuations shift and broaden the spectra, allowing for a more realistic description of the spectral line shapes. We observe that occasionally, BB transitions with low oscillator strength enter in the near-UV window due to mild energy fluctuations captured by EHEF. Instead, extremely large fluctuations are observed for individual cases with energy shifts as large as 3 eV. This finding shows the limitations of the EHEF method, which occasionally may overestimate the effect of electrostatic fluctuations. For this reason, we decided to exclude these states from the simulations and to focus on the sole Tyr and Phe residues. The Tyr–Tyr, Phe–Phe, and Tyr–Phe coupling, which are negligible with respect to the system–bath coupling, were set to zero. The spectrum based on the MD simulations (Figure 5b) resembles the main features of the single snapshot (Figure 5a). Notably, it exhibits diagonal peaks with an elliptical nature, which is a signature of the introduced static disorder.

Using the same criterion for “strong” site mixing introduced for the cYY dipeptides (i.e., no site coefficient in the excitons should have a value > 0.9), we find that only in about 2.5% of the snapshots (five out of 200 snapshots) did the Tyr residues mix strongly and form pair-wise delocalised states. The majority of the snapshots exhibit highly localised states after diagonalisation of the Hamiltonian (coefficients > 0.999). Therefore, we expect the pulse polarisation combination xxxx–3xxyy 2D technique to display rather weak signals, with a line shape that strongly resembles that of the non-degenerate, weakly mixing chromophores.

Analysis of the 2D near-UV signals for the cPY and cYY peptides showed that the most intense diagonal signal was to the L_b_(*u*_1_, *v*_0_) state, which is in line with the intensities of the near-UV CD bands for, e.g., cPY_300 and cYY_300_60, which are considered the most likely conformers. Moreover, the second and third strongest bands in the near-UV CD for cYY_300 and cYY_300_60 are due to the L_b_(*u*_0_, *v*_0_) (286.0 nm) and L_b_(*u*_2_, *v*_0_) (273.5 nm) transitions, which are the second and third most intense diagonal signals in the 2D near-UV spectra. These three transitions are also dominant in the near-UV CD and 2D spectra for BPTI. In addition, the 2DUV spectra for BPTI features a weak signal around 37,500 cm^−1^, which is associated with the L_b_(*u*_1_, *v*_1_) state of Tyr (at 37,365 cm^−1^). The EHEF corrected spectrum for BPTI, using an average over 200 MD snapshots, features elliptical peaks on the diagonal compared to the non-EHEF corrected spectrum. In general, EHEF is an effective way to efficiently account for electrostatic fluctuations, but unrealistic extremely large fluctuations are observed from time to time. Improving EHEF may constitute an important direction to follow to improve the ab initio simulation of spectra. Only 2D maps with a waiting time *t*_2_ = 0 were considered here: a wealth of additional information may be obtained by looking at *t*_2_ > 0, where system-specific variations of the signals intensity and shape over different time scales may be observed. The simulation and analysis of the information content of non-zero *t*_2_ 2D maps will be explored in future publications.

## 3. Methods

Avogadro [42] was employed to construct the geometries for the three cPY and six cYY model conformers. These model conformers are named according to their χ_1_ dihedral angles(s) (in degrees) for the Tyr residue(s) (N-C_a_-C_b_-C_g_). DichroCalc [43] was used to compute the CD spectra for the cyclic dipeptide and BPTI. The software implements the exciton framework, with the transition densities associated with excitations of biological chromophores represented by a large number of point charges (monopoles) that reproduce the electrostatic potential arising from the density. We used ab initio parameters for the amide backbone [44] and ab initio parameters with vibrational fine structure for the aromatic side chains of Phe and Tyr [18]. The vibrational transitions are incorporated by extending the exciton Hamiltonian; the electric transition dipole moments and the monopoles are scaled by a normalised Franck–Condon overlap integral. Additional details have been described previously [18,19].

All 2DUV simulations were performed with SPECTRON [45], using the same parameters as for the CD calculations. The original Hamiltonian was obtained from the DichroCalc program and contains the excitation energy and coupling between different residues. The vibrational fine structure of the L_b_ states of Tyr and Phe, the former being strongly coupled to a ring breathing mode at 800 cm^−1^ with reorganisation energy of ca. 990 cm^−1^, the latter to two intra-molecular modes at 570 and 980 cm^−1^ with reorganisation energies of 300 and 660 cm^−1^, respectively, was incorporated by means of site-specific spectral densities $J\left(\omega \right)=\underset{i}{\sum }\omega_{i}^{2}\lambda_{i}^{2}\delta \left(\omega -\omega_{i}\right)$ where $\omega_{i}$ and $\lambda_{i}$ are the frequency and reorganisation energy of the *i*th mode.

The rigorous treatment of vibronically coupled dimers/multimers, i.e., of systems in which neither the inter-site (excitonic) coupling V nor the coupling with intra-molecular vibrations λ can be treated perturbatively, as in the limiting cases of Redfield (λ << V) and Förster (V << λ) theories, poses a challenge for simulation. Proper treatment would require either to consider full vibronic Hamiltonians or to account for the inter-exciton spectral densities in an appropriate way [46]. Since neither of the two options is currently available in SPECTRON, we adopted two different strategies: as in the system under study, the couplings between the residues are small compared to the intra-molecular vibrational coupling, the Förster regime is an adequate approximation for simulating parallel polarised 2D maps, which are insensitive to very weak exciton couplings. For this reason, the inter-site (exciton) couplings between Tyr–Tyr, Phe–Phe, and Tyr–Phe residues were set to zero in the simulations of the full vibronic spectra (Figure 2 and Figure 5).

In contrast, crossed polarised xxxx–3xxyy 2D maps (Figure 3) are extremely sensitive to the coupling network between the chromophores: the signal is vanishing in case of zero coupling, and it is different from zero otherwise. To describe these subtle effects properly, exciton couplings have to be considered. To facilitate this within the framework of sites strongly coupled to vibrational degrees of freedom, the following strategy was adopted: instead of considering the electronic L_b_ state of Tyr in the Hamiltonian, and to account for its intra-molecular vibrational coupling via a spectral density, we directly focused our attention on the sole L_b_(*u*_0_, *v*_0_) vibronic transition, performing 2DUV simulations only on a narrow window around its absorption energy. Experimentally, this can be realised by a set up in which the spectral bandwidth of the pulses is sharply tuned to the GS-L_b_(*u*_0_, *v*_0_) transition.

To account for the homogeneous broadening of the spectra due to the environment, all transitions were coupled to a Drude–Lorentz-type spectral density with reorganisation energy of 300 cm^−1^ (for all the studied systems) and a cutoff frequency of 85 cm^−1^. For the simulations of the cYY dipeptides with static disorder in 500 realisations, the site energies of the chromophores (diagonal elements in the Hamiltonian) were replaced by values randomly selected from a Gaussian distribution centered at the Tyr excitation energy with a 100 cm^−1^ variance.

The SPECTRON code takes the site excitation energies, site–site coupling strength (in the form of an excitonic Hamiltonian), site transition dipole moments, and site spectral densities and computes (in case of non-zero coupling) exciton states and properties (exciton transition dipole moments and exciton coupling to the spectral densities). Additional parameters are the system temperature (set to 300 K), the polarisation pulse set ups (parallel—xxxx- and cross-polarised—xxyy-). Transport between excitons was neglected (due to the very small coupling between sites): by virtue of this assumption, the line-shape of the obtained 2DUV maps is exact [46]. A concise summary of the relation between ab initio data and 2DUV spectra is being reported elsewhere [47]. A sample of the SPECTRON input used is given in the SI.

MD simulations on BPTI used an X-ray (PDB entry 5PTI) and 20 NMR models (PDB entry 1PIT) as starting structures. For each structure, production dynamics were run for 10 ns from which 500 snapshots were extracted. Initial structures of BPTI were taken from the Protein Data Bank (PDB) and solvated using CHARMM-GUI [48] in a truncated octahedral box of around 4600 TIP3P water molecules [49]. After minimisation and equilibration, production simulations with a 2 fs timestep were performed with periodic boundary conditions in the NpT ensemble using NAMD [50]. All bond lengths involving hydrogen atoms were fixed using the SHAKE algorithm [51]. CD spectra were computed for all snapshots, and the 200 snapshots with the CD closest (based on RMSD) to the experimental spectrum [36] were selected for the calculation of 2DUV spectra.

Interactions of a chromophore with the surrounding electrostatic field can shift electronic excitation energies considerably. The transition energy is affected by the fluctuating electrostatic potential arising from the solvent and the rest of the protein. The variability of the transition energy (the diagonal term in the exciton Hamiltonian) can have a significant influence on 2DUV lineshapes. Thus, to explore the impact of this, we used the EHEF correction developed by Jiang et al. [41].

## Figures and Tables

**Table 1:** Experimental [34,35] and computed [34,38] electronic transition energies (Δ*E*) and oscillator strengths (*f*) of the singlet excitations of phenol, which were labeled following Platt’s notation.

Method	L_b_ Δ*E*/eV (*f*)	L_a_ Δ*E*/eV (*f*)	B_a_ Δ*E*/eV (*f*)	B_b_ Δ*E*/eV (*f*)
EOM-CCSD/aug-cc-pVTZ + R [35]	4.95 (0.021)	6.16 (0.034)	7.01 (0.389)	7.10 (0.517)
CASPT2 [38]	4.54 (0.005)	5.66 (0.014)	6.30 (0.797)	6.49 (0.690)
Expt [34]	4.59 (0.020)	5.82 (0.132)	6.70 (0.636)	6.93 (0.467)
Expt [35]	4.61	6.00	6.74	6.74

**Table 2:** Analysis of 2DUV data reported in Figure 3 for the three systems cYY_300_300, cYY_300_60, and cYY_60_60. A modified cYY_60_60 (labeled Mod. cYY_60_60) was also considered, in which the transition dipole moments (TDMs) of the Tyr residues were forced to have a 70° angle between them. xxxx–3xxyy signal intensities smaller than 0.1% have been set to zero. The reported coupling strength is the Franck–Condon weighted electronic coupling between the L_b_(u_0_) Tyr vibronic transitions. Strong mixing is defined as follows: the exciton wave functions must have coefficients between 0.71/±0.71 (perfect exciton) and 0.90/± 0.44.

	cYY_300_300	cYY_300_60	cYY_60_60	Mod. cYY_60_60
Tyr–Tyr TDM angle	70°	70°	10°	70°
Tyr–Tyr coupling strength	0.4 cm^−1^	2.6 cm^−1^	7 cm^−1^	7 cm^−1^
**Degenerate Sites (strong state mixing)**	**xxxx–3xxyy Signal Intensity**			
% with respect to xxxx	9.7%	6.3%	0.3%	6.6%
**Static Disorder (500 snapshots)**				
% with respect to xxxx	0%	0.6%	0.1%	1.9%
% strong mixing configurations	1–2%	4–5%	10–11%	10–11%

## Data Availability

The data presented in this study are available on request from the corresponding author.

## Supplementary Materials

The following are available online, Figure S1: Real 2DUV spectra in the near UV for the Mod cYY_60_60 conformer in the degenerate sites, non-degenerate sites, and static disorder cases, Figure S2/S3: Real and Imaginary 2DUV spectra in the near UV for the three conformers of cYY, in both the degenerate sites (S2) and the static disorder (S3) cases, employing the xxxx, 3xxyy, and xxxx–3xxyy polarisations, Figure S4: Real and Imaginary 2DUV spectra in the near UV for BPTI averaged over 200 MD snapshots, employing xxxx polarisations. Figure S5: representative SPECTRON input used to compute the 2DUV spectra reported in the paper.

## Sample Availability

Samples of the compounds are not available from the authors.
