# Supplementary material for: Near-Ultraviolet Circular Dichroism and Two-Dimensional Spectroscopy of Polypeptides
Source: Molecules. 2021 Jan 13;26(2):396. doi: 10.3390/molecules26020396 (PMC7828623; doi:10.3390/molecules26020396)
Supplement: Supplementary file 1 [file molecules-26-00396-s001.pdf]

molecules

MDPI

Near-Ultraviolet Circular Dichroism and Two-Dimensional Spectroscopy of Polypeptides

Francesco Segatta [1], David M. Rogers [2], Naomi T. Dyer [2], Ellen E. Guest [2], Zhuo Li [3], Hainam Do [4], Artur Nenov [1], Marco Garavelli [1] and Jonathan D. Hirst [2,*]

[1] Dipartimento di Chimica Industriale "Toso Montanari", Universita' degli Studi di Bologna, Viale del Risorgimento, 4, I - 40136 Bologna, Italy; francesco.segatta2@unibo.it (F.S.); artur.nenov@unibo.it (A.N.); marco.garavelli@unibo.it (M.G.)
[2] School of Chemistry, University of Nottingham, University Park, Nottingham, NG7 2RD, UK; david.rogers@nottingham.ac.uk (D.R); naomi_414@hotmail.com (N.D.); ellen.guest@nottingham.ac.uk (E.G.)
[3] School of Pharmaceutical Sciences, Jilin University, Changchun 130021, China; zhuoli198602@gmail.com
[4] Department of Chemical and Environmental Engineering, University of Nottingham Ningbo China, Ningbo 315100, China, & New Materials Institute, University of Nottingham Ningbo China, Ningbo 315042, China; hainam.do@nottingham.edu.cn
* Correspondence: jonathan.hirst@nottingham.ac.uk

S1. 2DUV Mod cYY_60_60

Real 2DUV spectra with xxxx–3xxyy polarisation for the **Mod cYY_60_60** conformer are compared in Figure S1 for **degenerate sites, non-degenerate sites,** and the **static disorder** cases. Note how the spectral shape resembles that of the cYY_60_60 maps while being much more intense.

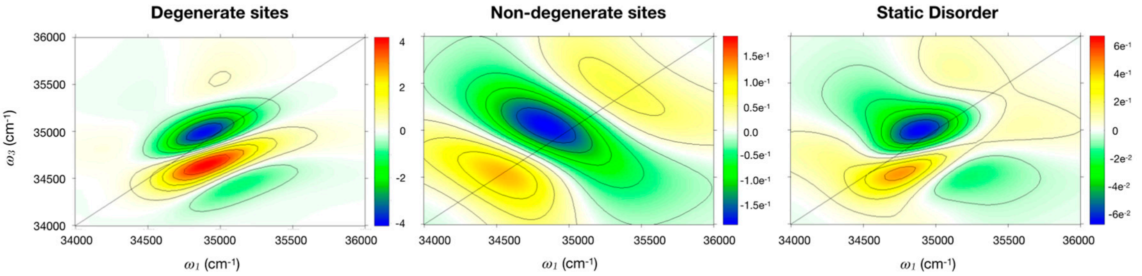

Figure S1. (Real) 2DUV spectra with xxxx–3xxyy polarisation for Mod cYY_60_60 conformer are compared in the case of degenerate sites and, consecutively, strong wave function mixing (a), for non-degenerate sites (very weak/null wave function mixing) (b), and for static disordered (weak wave function mixing) spectra (c). The intensity has been renormalized to the number of Tyr, and, in the case of static disorder spectra, also for the number of snapshots (500).

S2. 2DUV of cYY_300_60

Real and Imaginary 2DUV spectra with xxxx, 3xxyy, xxxx–3xxyy polarisation for the cYY_300_60 conformer are compared in Figure S2 for the **degenerate sites** case reported in the main text. Note how the xxxx and 3xxyy cases look very similar, and that only thir combination (xxxx–3xxyy) is able to highlight subtle differences between the two polarisations setups. As already noted in the main text, the highly structured spectra reported for the **degenerate sites** case are

dramatically modified in the presence of **static disorder**, which decreases the amount of configurations with "strong wave function mixing" and produces an average of the degenerate and non-degenerate spectra (see Figure S3).

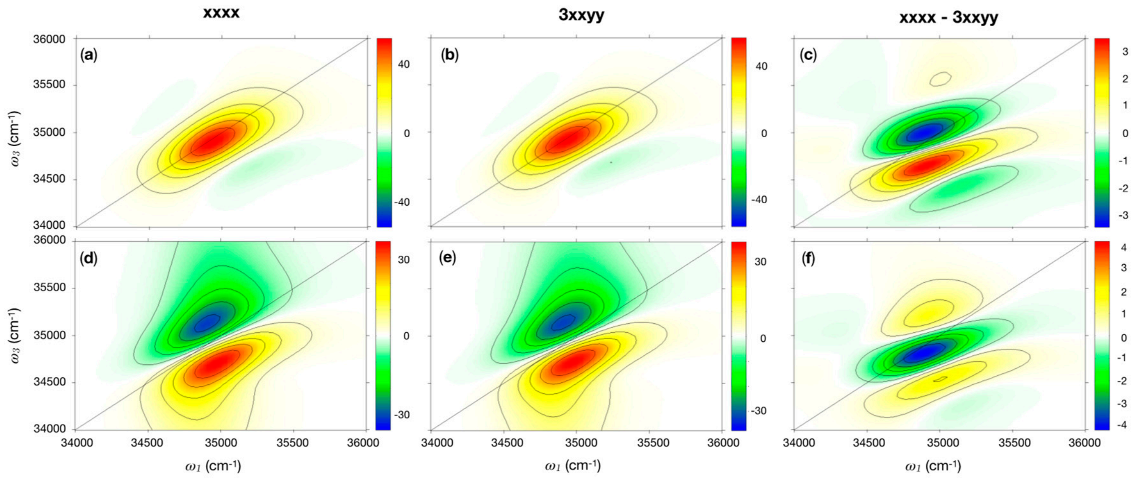

Figure S2. Real (a-c) and imaginary (d-f) 2DUV spectra with xxxx, 3xxyy, xxxx–3xxyy polarisation for the cYY_300_60 conformer in the **degenerate sites** case. The intensity has been renormalized to the number of Tyr.

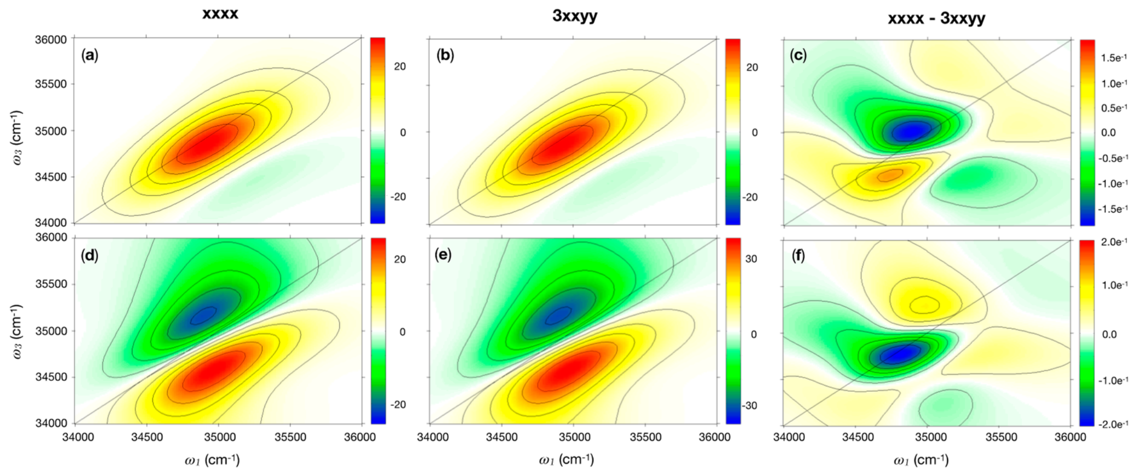

Figure S3. Real (a-c) and Imaginary (d-f) 2DUV spectra with xxxx, 3xxyy, xxxx–3xxyy polarisation for the cYY_300_60 conformer in the **static disorder** case (500 snapshots, variange of 100 cm^{-1}). The intensity has been renormalized to the number of Tyr and the number of snapshots.

S3. 2DUV of BPTI

Real and imaginary 2DUV maps for the xxxx polarisation are reported for a single snapshot and the average spectrum from the 200 MD snapshots of the PBTI system.

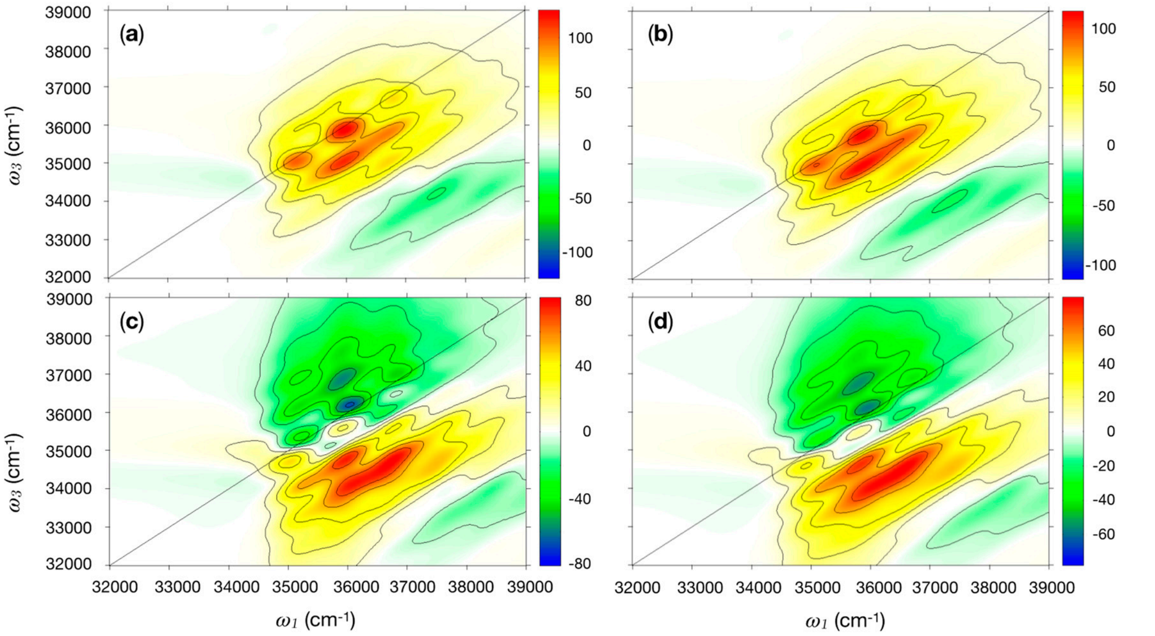

Figure S4. Real (a-b) and imaginary (c-d) 2DUV spectra with xxxx polarisation for the single snapshot with degenerate sites (a,c) and the 200 MD snapshots (b,d) of the BPTI system. The intensity has been renormalised to the number of Tyr and by the number of snapshots (in (b) and (d)).

S4. Representative 2DUV Spectron Input File

As described in the main text, two strategies for simulating 2DES spectra were employed:

1. Full vibronic spectra are shown in Figures 2 and 5 of the main text (and Figures S4 in the SI) with xxxx polarization. These were computed by coupling the Tyr L_b states to its dominant intra-molecular vibration mode, which is characterised by a frequency of 800 cm^{-1} and reorganisation energy of ca. 990 cm^{-1}. All couplings between Tyr chromophores were set to zero in the exciton Hamiltonian.
2. A single vibronic state, namely $L_b(u_0, v_0)$, was considered when the polarisation combination xxxx–3xxyy was simulated (Figure 3 in the main text, and Figures S1–S3 in the SI). The coupling between $L_b(u_0, v_0)$ and the other transitions were kept as obtain in DichroCalc. Since we are considering a single vibronic state, no coupling to the intra-molecular mode spectral density was considered here.

The complete treatment of vibronic dimers/multimers requires either considering full vibronic Hamiltonians or to account for the inter-exciton spectral densities in an appropriate way [1]. The spectra were simulated with the Spectron code [2,3]. We report hereafter the input file that were used to simulate 2DUV spectra with both methods (1) and (2). Note that the input file for method (2) will differ with respect to that of method (1) mainly by the presence of a single spectral density coupled to all the states, which is the Drude–Lorentz type spectral density with a reorganisation energy of 300 cm-1 (for all the

studied systems) and cutoff frequency of 85 cm-1 to describe the interaction between the given state and the environment. The coupling with the intra-molecular mode is removed for method (2) (as we are considering a specific vibronic state).

```
$REGISTRATION
PP
$END

$PP
CAL_METHOD       SOS_CGF_C

INI_FREQ1        30000.0
FIN_FREQ1        41000.0
NUM_FREQ1        250
INI_FREQ3        30000.0
FIN_FREQ3        41000.0
NUM_FREQ3        250

DEL_TIME2        0
NUM_SHOTS        1

OPT_POL1         1 0 0
OPT_POL2         1 0 0
OPT_POL3         1 0 0
OPT_POL4         1 0 0

OUT_FILE         sig-2DUV-xxxx.dat
$END

$SYSTEM
SYSTEM_KEYWORD   Excitonic_Disordered_ens__

NUMMODES         8
INP_HAM_L_       Hamil.dat
INP_DIP_L_       Edipl.dat
ELECTRONIC       1

TRANSPORT        1
TRANSPORT_RATES  transport_rates.txt

DISORDER_INTRA_DIAG_GAUSS 0
SYSTEM_BATH_COUPLING_MM coupling_file
$END

$BATH
OSCILLATORS_NUM     3
TEMPERATURE         300
BATH_MODEL          MM_Continuous_spectral_density
SPECTRAL_DENSITIES  spectral_densities.txt
$END
```

Figure S5. Spectron input file for 2DUV spectra simulated within method (1) for BPTI. In the REGISTRATION section, one specifies the type of signal to compute (PP stands for 2DES pump probe spectrum). The PP section specifies the window and the number of samples per each frequency axis, the time t_2 (set to zero here), the polarisation setup, and the name of the output file in which the spectrum will be saved (sig-2DUV-xxxx.dat). The method with which the spectra are computed is named SOS_CGF_C, which stands for sum-over-states and cumulant (expansion) of Gaussian fluctuations "C" (which allows for dynamic Stoke shift to be considered). In the SYSTEM section, information about the system is given: one specifies that the excitonic Hamiltonian (contained in the Hamil.dat file) has eight rows (and columns). ELECTRONIC 1 specifies that each site is a two-level system. In the Edipl.dat file, transition dipoles of the sites are given, while the transport_rates.txt file contains the rates between the states (that we assume to be zero here for simplicity). The couplings of the sites to the various spectral densities are specified in the coupling_file file. In the BATH sections, the system-bath properties are listed. The temperature of the system is set to 300 K, and three spectral densities are considered: one that gives the coupling with the environment, modeled through an Drude–Lorentz-type spectral density, while two others that specify the couplings of Tyr and Phe to their intra-molecular modes (note that within method (2), only the first spectral density is

considered). Spectral densities are given as numerical functions (MM_continuous_spectral_density keyword) and specified, one after one, in the spectral_densities.txt file.

References

1. Kühn, V.; May, O. Charge and Energy Transfer Dynamics in Molecular Systems. Wiley-VCH Verlag GmbH & Co. KGaA, Weinheim, Germany, 2011, doi:10.1002/9783527633791.
2. Zhuang, W.; Abramavicius, D.; Hayashi, T.; Mukamel, S. Simulation protocols for coherent femtosecond vibrational spectra of peptides. J. Phys. Chem. B, **2006**, *110*, 3362–3374, doi:10.1021/jp055813u.
3. Segatta, F.; Nenov, A.; R. Nascimento, D.; Govind, N.; Mukamel, S.; Garavelli, M. iSPECTRON: A simulation interface for linear and nonlinear spectra with ab-initio quantum chemistry software, *submitted to J. Comp. Chem*.
